# Human Body Parts Proximity Measurement Using Distributed Tactile Robotic Skin

**DOI:** 10.3390/s21062138

**Published:** 2021-03-18

**Authors:** Jan Klimaszewski, Michał Władziński

**Affiliations:** 1 Warsaw University of Technology, Faculty of Mechatronics, Institute of Automatic Control and Robotics, A. Boboli 8 St., 02-525 Warsaw, Poland; 2 Warsaw University of Technology, Faculty of Mechatronics, Institute of Metrology and Biomedical Engineering, A. Boboli 8 St., 02-525 Warsaw, Poland; michal.wladzinski@pw.edu.pl

**Keywords:** proximity sensor, electronic skin, robotic skin, distributed sensor, flexible electronics, real-time, graphene nanoplatelets, tactile sensor

## Abstract

Safety in human–machine cooperation is the current challenge in robotics. Safe human–robot interaction requires the development of sensors that detect human presence in the robot’s workspace. Detection of this presence should occur before the physical collision of the robot with the human. Human to robot proximity detection should be very fast, allowing machine elements deceleration to velocities safe for human–machine collision. The paper presents a new, low-cost design of distributed robotic skin, which allows real-time measurements of the human body parts proximity. The main advantages of the proposed solution are low cost of its implementation based on comb electrodes matrix and real-time operation due to fast and simple electronic design. The main contribution is the new idea of measuring the distance to human body parts by measuring the operating frequency of a rectangular signal generator, which depends on the capacity of the open capacitor. This capacitor is formed between the comb electrodes matrix and a reference plate located next to the matrix. The capacitance of the open capacitor changes if a human body part is in vicinity. The application of the developed device can be very wide. For example, in the field of cooperative robots, it can lead to the improvement of human–machine interfaces and increased safety of human–machine cooperation. The proposed construction can help to meet the increasing requirements for cooperative robots.

## 1. Introduction

Robotic manipulators have been widely used in industry, medicine, entertainment, etc., for over 50 years. In recent years, cooperative robotics have been designed to facilitate human–machine cooperation and introduce robots to the direct human space while maintaining safety rules. To achieve these goals, it is necessary to develop robots which can detect human presence before physical collision. It will allow the machine to decelerate its elements to velocities safe for collision with human. To achieve this, and to be useful for the safety system of the collaborative robot, the proximity sensor must be very fast.

This paper presents a new, low-cost design of artificial robotic skin, which allows real-time measurements of the human body parts proximity. This goal is achieved by measuring the distance to a part of the human body by measuring the operating frequency of a rectangular signal generator. The operating frequency changes with the change of the capacitance of the open capacitor formed between the comb electrode array and the reference plate placed next to the matrix. When a part of the human body is near the open capacitor, its capacity changes. In addition, the sensor is flexible, which allows it to cover most of the robot’s surfaces. At the current stage of project development, the sensor can operate in two modes: the touch measurement mode or the proximity measurement mode.

The most important advantages of the developed solution are the low cost of its production based on a matrix of comb electrodes printed on a flexible foil and a very fast measurement time, which was achieved thanks to a simple electronic design. An additional advantage of the developed sensor in the face of the development of collaborative robotics and human-machine interfaces is that the solution we propose supports a distributed approach in the implementation of touch and proximity sensors. This enables large and complex machine surfaces to be covered at low cost. The use of a digital system in the form of a rectangular signal generator additionally immunizes the measurement system against analog disturbances.

As part of the research, a prototype proximity sensor with measurement electronics hardware was designed and manufactured. The implementation of appropriate data processing algorithms was performed and the entire system was tested. The test results confirm the effectiveness of the developed device and methods.

The work layout is as follows. In Section 2, an overview of papers on proximity sensing is presented. In Section 3, a new proximity sensing device is presented, and the measuring system is briefly described. In Section 4, the measurement results of the developed prototype are presented. Finally, in Section 5, the test results are discussed and summarized.

## 2. Related Research

The robotic skin presented in the manuscript was screen-printed on the flexible film [1]. It can function in two modes: measuring the location and value of pressure from the touch, and measuring the proximity of human body parts. The manuscript mainly covers proximity measurement, however, for a better understanding of the subject, an overview of similar solutions for touch measurement is also provided.

In the field of touch measurement, there are many publications describing flexible devices for measuring the value and location of touch pressure [2,3,4,5,6]. The authors of Reference [7] broadly describe the development of the technology of producing flexible printable electronics over the years. A general overview of the application and classification of operating methods of this type of devices can be found, among others, in References [8,9]. Touch measurement is often performed as: resistance measurement [10,11], capacitance measurement [2,3], air pressure measurement [4], using the Hall effect [5]. Touch can also be measured using piezoresistivity, piezoelectricity [8] or with the use of methods combining the advantages of different approaches [6].

Particularly noteworthy is Reference [6]—it describes a sensor with a design that takes into account, as in our work, the use of a resistance matrix based on graphene. The touch measuring device described in Reference [6] combines a system based on resistance and capacity. The robotic skin that we have developed is low-cost, and its construction is based on graphene nanoplatelets. In the field of low-cost electronic skin production, dynamic development is still taking place. For example, the use of new materials (hydrothermally grown ZnO nanorods) to solve a similar problem can be found in Reference [12].

Reference [13] presents the measurement of touch with the use of a two-layer surface based on the measurement of capacity. A similar approach using inkjet printing technology is presented in Reference [14]. In Reference [10], the advantages of resistance sensors for touch measurement were highlighted. In Reference [11], the authors describe the application of this type of sensors for soft robotic grippers.

The structure of the resistor matrix for touch measurement and the measurement concept similar to that used by the touch measurement device presented by us can be found, in particular, in References [15,16,17].

Other interesting articles on robotic skin are References [18,19], which describe the elastic, stretchy skin and the measurement of the pressure position using only five electrodes.

An interesting approach to solve the problem of the robotic skin construction with multi-axis pressure measurement is the use of capacitive sensors [20,21]. Unfortunately, these articles do not contain measurement electronic system description.

The authors of Reference [22] describe a capacitor matrix for measuring multi-axis touch forces. The structure of the presented matrix is spatial (tumors and bumps) and there is a matrix of flat sensors spread on it. The described application of the device are the fingers of a robotic hand.

Proximity sensors are used in many devices from cars to industrial equipment and robots. When people work near industrial robots, the safety is imperative. It can be achieved by means of devices that detect the presence of humans in their surroundings.

There are many types of sensors that can detect the proximity of a physical object. Proximity sensors use many physical properties, such as light reflection [23,24,25,26], sound wave reflection [27,28,29,30,31], reflection of an electromagnetic wave in the radar [32,33,34], and also detecting changes in the selected parameter in the space around the sensor, such as electric permeability [35,36,37,38,39], magnetic permeability [38], or temperature [25].

The first group are reflection sensors. These sensors include transmitters that emit a signal to the surrounding space and receivers for detecting the returning signal.

Optical proximity detectors detect nearby objects by reflecting light from the object. Due to the background noise, near infrared light is used. Infrared is preferred over visible light in proximity sensing as the former is invisible and has lower background noise from indoor lighting or outdoor sunlight. Furthermore, a light scattering is proportional to the inverse of the fourth power of a wavelength; therefore, reflected light is much stronger for infrared for the same incident intensity. Light detectors are placed around the light emitter to detect light reflected from the object. By measuring the current in the photodetectors, the presence of the object [23] is detected. Detection of the presence of an object in the vicinity can be performed with the use of optical rangefinders placed in integrated circuits, like TCND5000 [26]. Time-of-flight measurement is also used in ultrasonic sensors. Due to the much lower speed of propagation of the sound wave, systems of this type are much simpler and can be made with the use of a simple microcontroller, e.g., the HC-SR04 [29].

The second group of sensors are systems that change their output signal as a result of the appearance of objects with appropriate properties in their surroundings. The most popular solutions use differences of permittivity between a detected object and the environment [35,36,37,38,39].

The appearance of an object in close proximity to the capacitor plates causes a change in its capacitance, which is measured in various ways. A common way to measure capacitance is to connect a sinusoidal voltage to the capacitor and measure the current [35]. By calculating the impedance of the capacitor, the measured impedance is converted into the capacitance [37].

There are also integrated capacitance to digital converters, for example: AD7148 [36,39]. These systems allow to measure capacitance for a larger number of channels by measuring the charge in the capacitors with Δ−Σ analog to digital converters. Commercially available chips are able to measure capacitance for 8 sensors with an update rate of 25 ms. In this case, the design of the electrode is also of great importance, as it enables early detection of an object near the sensor [40].

Other techniques are used to detect objects with different magnetic permeability [38]. The technique uses changes in induction loop impedance due to the appearance of an object in its vicinity with a permeability different from the surroundings. These solutions are suitable for metal detection.

Due to the high inertia and significant detection time, a rarely used solution are measuring systems that test temperature changes near the sensor. For this purpose, highly-stable resistance sensors, e.g., PCS1.1302 [25] are used.

In the literature, solutions that combine many modalities in one measuring cell are presented, as well. References [24,39] combine proximity sensor systems with touch sensors. The solutions proposed there focus on the design and construction of a universal module (skin cell) with specific sensors in such a way that it is possible to combine them into larger structures. Such solutions enable detection of an object in a specific area of the space around the robot. A significant disadvantage of such solutions is that the multiplication of the number of modules significantly increases the price and reduces the overall speed of operation of such a sensor system. For example, a combination of 66 skin cells with a total area of 450 cm${}^{2}$ connected by high-speed gigabit ethernet interface allows for obtaining measurements within 4 ms (250 Hz) [24]. The solution proposed herein is adapted to the idea of building sensors in a distributed manner and is devoid of the aforementioned disadvantages.

## 3. Developed Design

The design of the developed device consists of several parts. An important hardware element is the robotic skin—an array of FSR (Force Sensitive Resistor) and comb electrodes with the electronic board for touch measurements (both described in the Section 3.1). The additional electronic circuit for proximity measurements described in the Section 3.2 performs the tasks related to generating a rectangular signal, the operating frequency of which changes with the capacity of the open capacitor. Further on, in Section 3.3, the measurements of operating frequency of a rectangular signal generator are described.

The sensor was manufactured at the Faculty of Mechatronics, Warsaw University of Technology. With regard to the production costs of the presented system, we estimated them as follows. The total cost of an array of FSR and comb electrodes is approximately 0.54 EUR (euro) for an area of 200 mm × 200 mm consisting of 16 × 16 cells. This cost includes the price of: two sheets of foil (approximately 0.26 EUR), silver-based conductive paint (approximately 0.21 EUR) and graphene-based materials (approximately 0.07 EUR). In addition to the proximity measurements, which is the main area discussed in the manuscript, it is necessary to construct an electronic circuit for proximity measurements at a cost of less than 1 EUR for one robotic skin surface (which can vary in size). For many such surfaces, one microprocessor system for acquiring proximity measurements can be used. The microprocessor system used costs approximately 12 EUR. The cost of an array of FSR and comb electrodes can be scaled linearly with increasing surface area. The remaining elements of the proximity measurement system have a non-linear impact on the total cost of the system. More complex systems are more expensive. In view of the above calculations, we believe that the total cost of the presented system is low.

### 3.1. Tactile Robotic Skin

**Figure 1:**
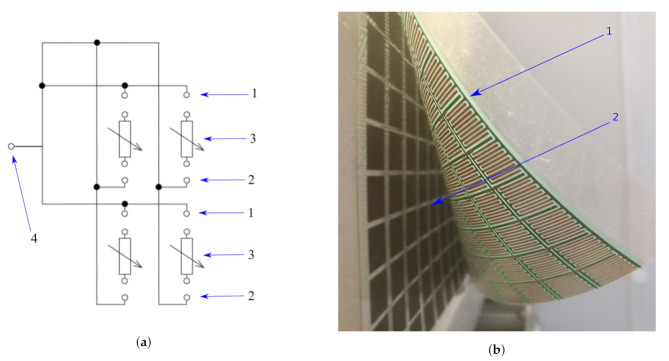
Tactile robotic skin. (**a**) Wiring diagram of robotic skin for proximity measurements for a 2 × 2 FSR matrix; 1—first connection of comb electrodes (electrodes connected in rows), 2—second connection of comb electrodes (electrodes connected in columns), 3—FSR sensors, 4—connection of open capacitor $C_{x}$ working plate described in Section 3.2. (**b**) the robotic skin; 1—a conductive layer of comb electrodes printed on plastic foil; 2—FSR sensors arranged in a rectangular pattern placed on a plastic foil.

The robotic skin described in this paper consists of two layers. The first one consists of a conductive layer of comb electrodes (marked as 1 in Figure 1b) printed on plastic foil and connected along columns and rows. The second one consists of FSR sensors arranged in a rectangular pattern (marked as 2 in Figure 1b) placed on a plastic foil. The FSR matrices have the size of a single sensor approximately 5 mm × 5 mm. In order to conduct the touch measurement, a matrix of FSR sensors with the dimensions of 16 × 32 cells was used. However, in order to present the measurement of the proximity, a matrix of FSR sensors with a size of 16 × 16 cells was used. Details of the matrix structure can be found in Reference [1]. For proximity measurement purposes, each of the columns and rows are connected to each other (Figure 1a) and act as open capacitor $C_{x}$ working plate described in Section 3.2.

Each resistive sensor is based on graphene nanoplatelets similar to that described in Reference [41]. In the described robotic skin, the FSR matrix cooperates with comb electrodes in order to detect the place and the contact force exerted thereon. The FSR matrix is not used for the proximity measurement—the electronic system described in Section 3.2 and Section 3.3 uses only comb electrodes for this purpose.

**Figure 2:**
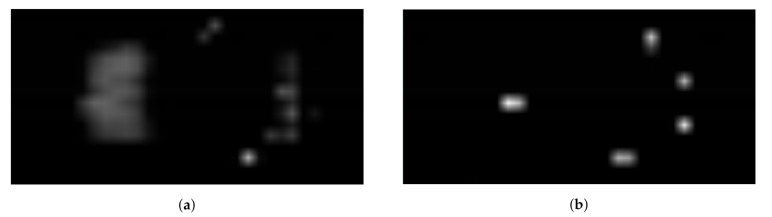
Example hand touch visualization for size of 16 × 32 cells. (**a**) Example hand touch. (**b**) Example fingertips touch.

In order to acquire touch measurements from robotic skin, a dedicated electronic measuring system and a PC application were developed. The measuring system communicates with a computer via simple serial communication and a USB port. The developed device acquires the pressure map from 16 × 32 FSR matrix at a rate of 48 frames per second, which is equivalent to one measuring cycle every 21 ms. Selected components affecting this speed and other details of the electronic touch measurement system can be found in Reference [1]. It is worth noting that the work described in this manuscript has managed to accelerate the measuring cycle of the touch measuring system by 9 ms (in Reference [1], the measuring cycle of 30 ms is described). An example of hand touch visualization is shown in Figure 2. Due to the relatively low resolution of the matrix, linear interpolation was used for the visualization of the system operation.

### 3.2. Rectangular Signal Generator

The proximity sensor is based on the electrical properties of the open capacitor. The capacitance of an open capacitor changes if an object with a relative permittivity other than vacuum permittivity is in proximity.

**Figure 3:**
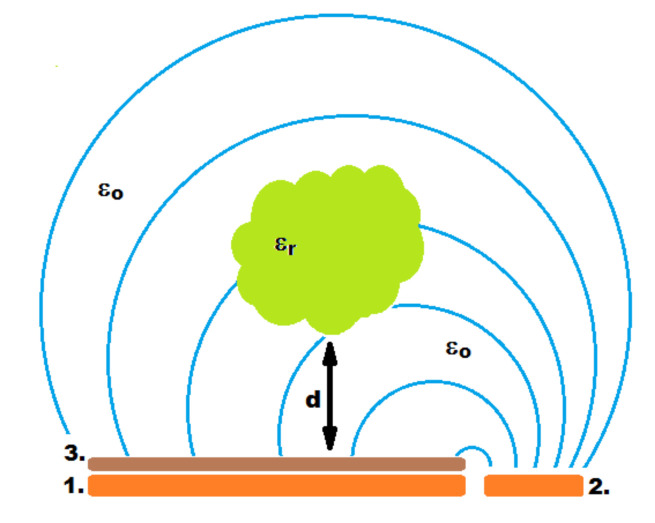
Principle of the proximity detector operation. 1—open capacitor working plate, 2—open reference capacitor plate, 3—resistive touch sensors, 4—objects with relative permittivity different than air.

The open capacitor is made of two plates: a working plate located under the resistance touch sensors and a reference plate located next to the touch sensors (Figure 3). If the object above moves to the work plate, the capacity of this set changes. When an object with relative permittivity different from air permittivity shifts towards the working plate, the capacity increases.

**Figure 4:**
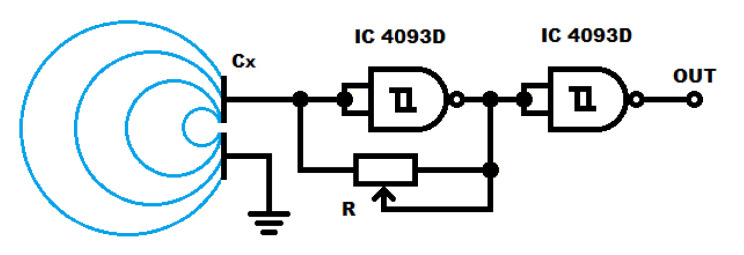
Electrical circuit of the measurement system for the change in capacitance of the open capacitor. $C_{x}$—open capacitor, R—adjusting potentiometer, IC (Integrated Circuit)—4093 Schmitt NAND gate.

The capacitance measuring system is based on a rectangular signal generator (Figure 4), in which operating frequency depends on the capacity of the open capacitor. The generator is implemented on CMOS NAND schmitt trigger gate, in which a capacitor is connected to the input, and a regulating potentiometer is placed in the feedback loop. The use of CMOS gates with high input resistance enables cooperation with a capacitor of small capacities. The adjusting potentiometer allows to set the basic frequency of generator operation.

In order to prevent the generator’s operating point from changing due to the output load, an additional gate separating the processor frequency measurement system has been added. The use of the IC 4093 CMOS gates allows the system to work with a voltage supply in the range from 2.5 to 18 V.

### 3.3. Microprocessor Measurement System

To measure the changes of the generator operating frequency the NUCLEO-F446RE microprocessor system with STM32 microcontroller at a clock rate of f=90 MHz was used. The output signal from the generator was connected to the appropriate digital inputs of microprocessor system. Setting the digital input in the external interrupt with rising edge trigger detection mode made it possible to measure the number of oscillation cycles *n* of the generator in a constant time $t_{c}=\frac{n_{c}}{f}=728.178$μs, where $n_{c}$ = 65,536 results from appropriate setting of the ARR (AutoReload Register), PSC (Prescaler), and CKD (Internal Clock Division) registers of the microprocessor counter. Due to the relatively high clock frequency of the microcontroller, it was possible to achieve a high accuracy of the time $t_{c}$ measurement and thus to achieve a good measurement accuracy of the frequency. This frequency is defined by the Equation (1).

(1) $$f_{g}=\frac{n}{t_{c}},$$

where:

$f_{g}$—oscillation frequency of the generator (MHz),

$t_{c}$—constant generator oscillation measurement time fixed at 728.178μs, and

*n*—the number of oscillations of the generator measured in the experiment.

The obtained measurement results are discussed in detail in the next section. It is worth emphasizing that the adopted method allows to obtain very fast measurement acquisition times. The time of acquisition of one measurement with clocking f=90 MHz and timer counter counting up to $n_{c}$ = 65,536 is approximately 728 μs.

## 4. Test Results

### 4.1. Experiment Description

**Figure 5:**
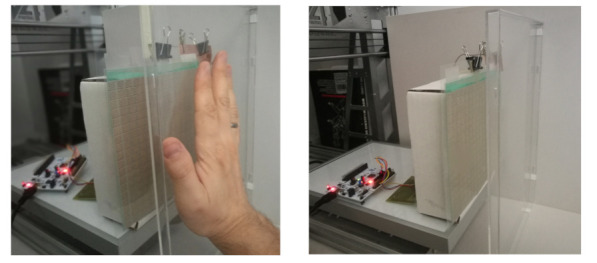
Illustrative presentation of the measuring setup, side view.

**Figure 6:**
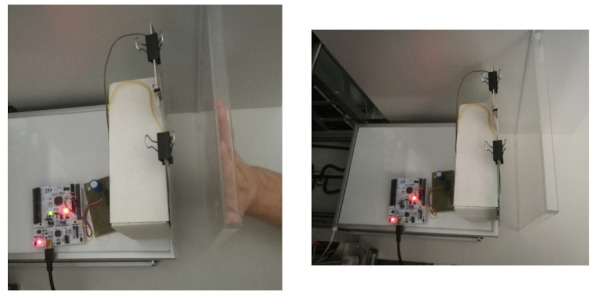
Illustrative presentation of the measuring setup, top view.

As part of the tests, an experiment was carried out using a CNC (Computer Numerical Control) type machine. Sample images of the test setup are presented in Figure 5 and Figure 6. The CNC machine table was modified and served as a support for the vertically placed comb electrode array along with the measuring system. A partition with a hand was placed vertically to the machine base and fixed to the table. The fixed partition was designed to keep the human hand in a fixed position throughout the experiment.

The performed tests can be divided into two experiments. The first experiment was to determine the characteristics of the proximity measurement of the presented measuring device, which is a robotic skin. In the second experiment, measurements were carried out to illustrate the effect of the measurement acquisition time on the accuracy of the proximity measurement.

The first experiment consisted of the acquisition of measurements from the system described in Section 3, while changing the position of the CNC machine table. As shown in Figure 3, during the experiment, the electrode array was placed at distance *d* ranging from 0 to 100 mm with the partition distance change in 1 mm steps. Approximately 10,000 of measurement points were recorded for each distance.

The second experiment concerned the proximity measurement times. As stated in the Section 3.2, the acquisition time of one measurement is approximately 728 μs. In order to extend the experiments, additional tests were performed to illustrate the operation of the system depending on various settings of the $n_{c}$ value. This value directly influences the measurement time. Illustrative tests were conduct using the hardware setup described at the beginning of this section. The tests course was that the hand was brought closer to the robotic skin twice so as to cause a noticeable change in the measurement value *n* described in the Equation (1). The actual ground truth distance between the hand and the robotic skin was not recorded during these illustrative tests.

### 4.2. Results Summary

**Figure 7:**
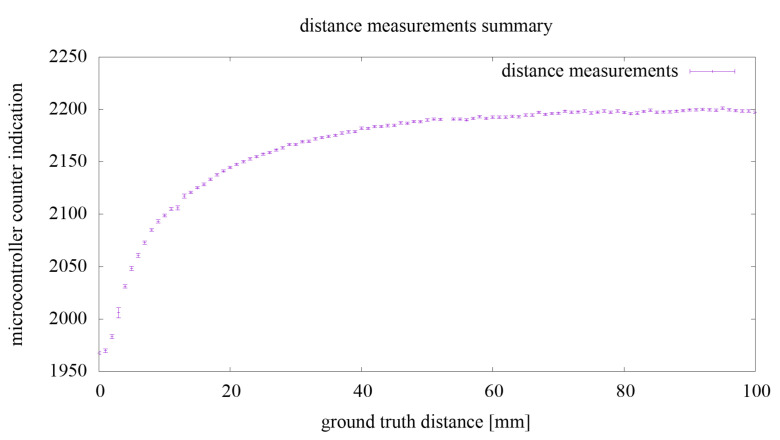
Chart summarizing the measurements carried out as part of the experiment.

Regarding the first experiment, the summary of the measurement results for the value of *n* from Equation (1) are shown in the chart in Figure 7. Each measurement point has a value equal to the average of all measurements for a fixed distance from the hand to the comb electrode array. For each measurement point, the measurement uncertainty, calculated as the standard deviation from the mean, is marked with a vertical line. The standard deviation from the mean at a given point for all measurements was 1.19.

As a result of the preliminary analysis, the measurement values (microcontroller counter indication) of *n* were acquired from the range around 1950–2200, which means changes in the value of the generator signal period in the range of 330–374 ns and changes in the generator frequency in the range around 2.67–3.02 MHz. This experiment results are discussed in the next subsection of the paper.

**Figure 8:**
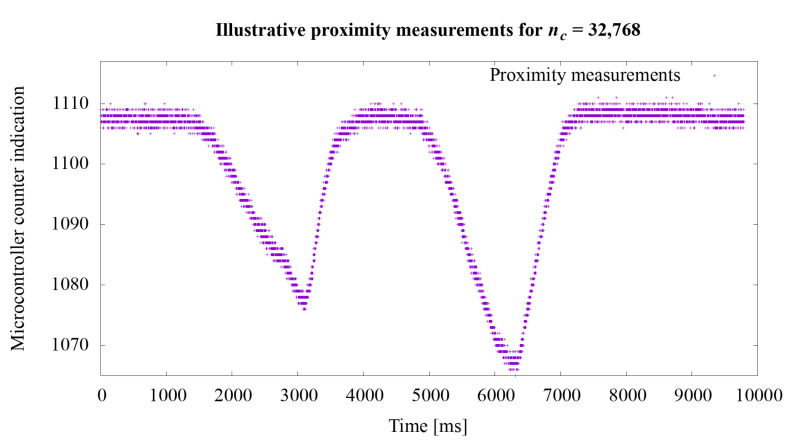
Illustrative proximity measurements for acquisition time 361 μs.

**Figure 9:**
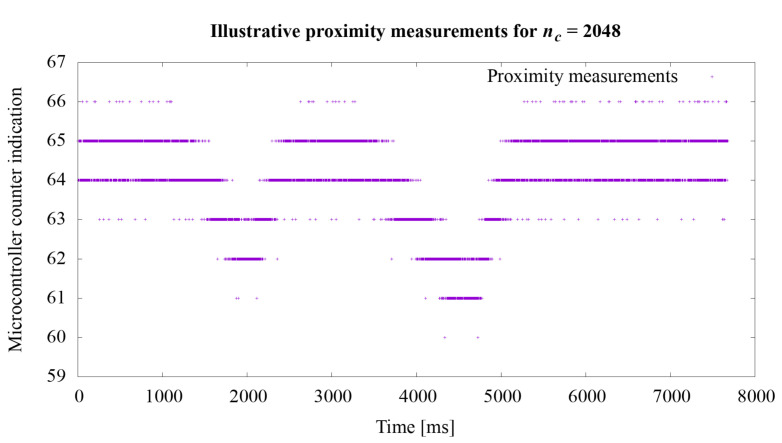
Illustrative proximity measurements for acquisition time 22.5 μs.

**Figure 10:**
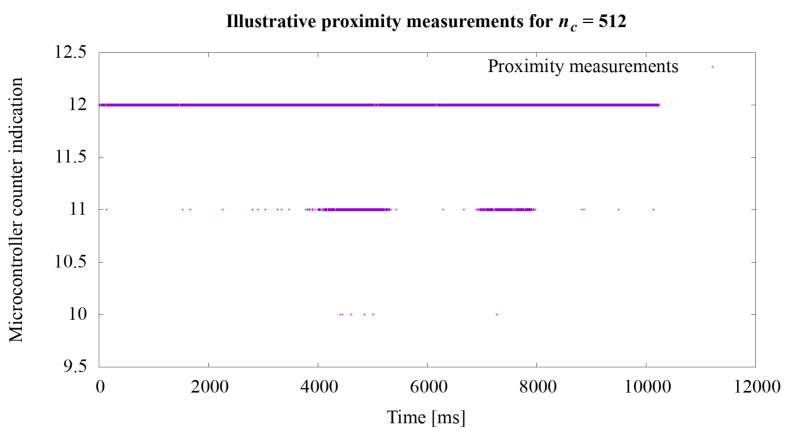
Illustrative proximity measurements for acquisition time 5.63 μs.

Regarding the second experiment, the measurement results for the value *n* from the Equation (1) are shown in the graphs in Figure 8, Figure 9 and Figure 10. In the first experiment, the value of $n_{c}$ directly influencing the measurement time was set at 65,536, which gave the acquisition time approximately 728 μs. The graphs show how the sensitivity of the system to object proximity changes due to the reduction of this time. For the $n_{c}$ value equal to: 32,768, 16,384, 8192, 4096, 2048, 1024, 512, 256, the measurement acquisition time is, respectively: 361, 180, 90, 45, 22.5, 11.26, 5.63, and 2.82 μs. On the basis of the selected graphs, it can be seen how the reduction of the measurement acquisition time has a negative effect on the proximity detection. A more extensive discussion of these results is provided in the next section of the manuscript.

### 4.3. Discussion

Regarding the first experiment, based on the measurements and the Equation (1), the values of the generator oscillation frequency corresponding to the measurements were determined. The frequency characteristic $f_{g}\left(d\right)$ (MHz) of the generator as a function of the distance *d* (mm) from the developed sensor was adjusted to these values. This characteristic is presented in Equation (2).

(2) $$f_{g}\left(d\right)=a-\frac{b}{{\left(d+c\right)}^{2}},$$

where:

a,b,c—characteristics parameters, and

*d*—hand distance from the developed sensor (mm).

**Figure 11:**
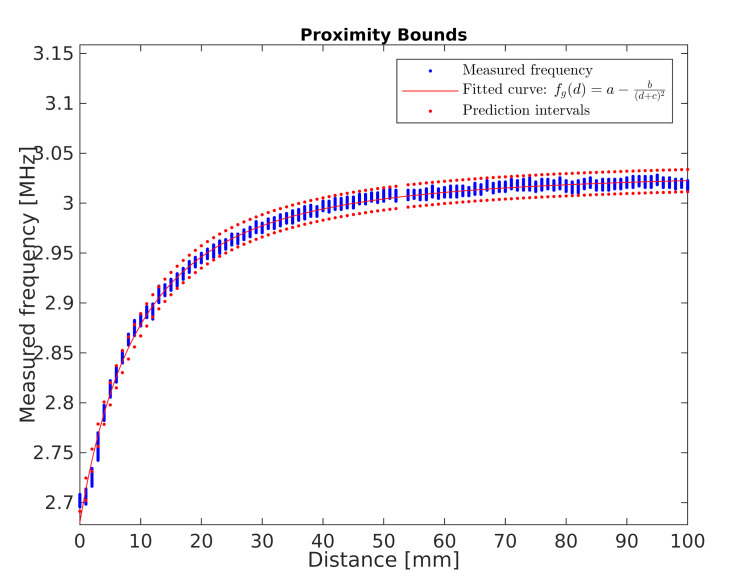
Measured frequency characteristic of the developed proximity sensor.

Equation (2) with measurement points is shown graphically in Figure 11. The a,b,c parameters and their 95% confidence bounds (lower and upper bounds in parentheses, respectively) were set at a=3.032(3.0319,3.0320),b=133.9(133.8355,134.0043),c=19.51(19.4991,19.5126). For this curve, SSE (Sum of Squares Due to Error) statistics equals to 19.441, R-Square equals to 0.9965, Degrees of Freedom Adjusted R-Square equals to 0.9965 and RMSE (Root Mean Squared Error) equals to 0.0043. Observation non-simultaneous curve prediction intervals are marked in red.

The developed sensor is to be used to determine human proximity. For this purpose, in further works, the inverse characteristic to that presented in Equation (2) was transformed. The inverse characteristic is represented by Equation (3). Using the transformed equation, it is possible to calculate the distance from the sensor by measuring the frequency of the signal.

(3) $$d\left(f_{g}\right)=\sqrt{\frac{b}{a-f_{g}}}-c.$$

**Figure 12:**
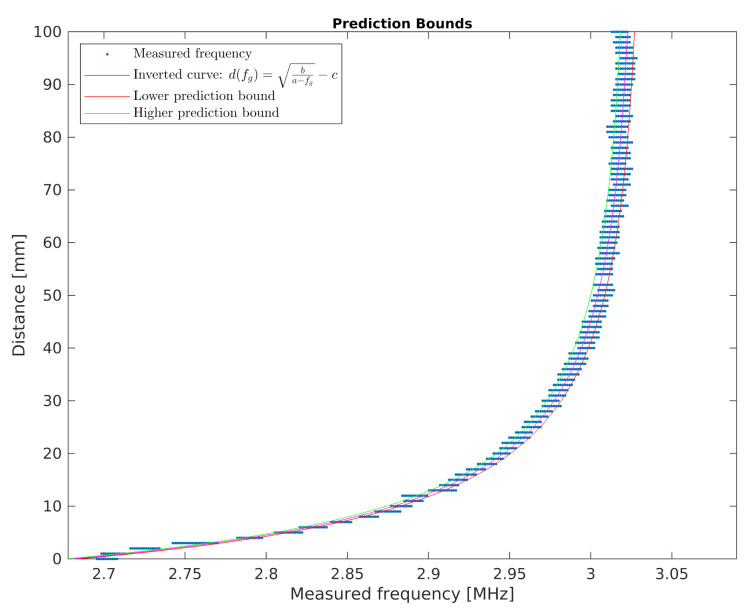
Inverse characteristic of the developed proximity sensor.

For the $d(f_{g})$ characteristic (Figure 12), the prediction intervals were estimated using the following procedure:

(1) For each value of $f_{gi}$ from the $f_{g}$ domain (with the $\frac{1}{t_{c}}$ step), the value of $d_{i}\left(f_{gi}+RMSE\right)$ and $d_{i}\left(f_{gi}-RMSE\right)$ was calculated. The values obtained are denoted in the chart in Figure 12 as lower and higher prediction bounds.
(2) For each value of $f_{gi}$, the prediction error $PE_{i}$ was determined according to the Equation (4).
  (4) $$PE_{i}=\frac{d_{i}\left(f_{gi}+RMSE\right)-d_{i}\left(f_{gi}-RMSE\right)}{2}.$$

**Figure 13:**
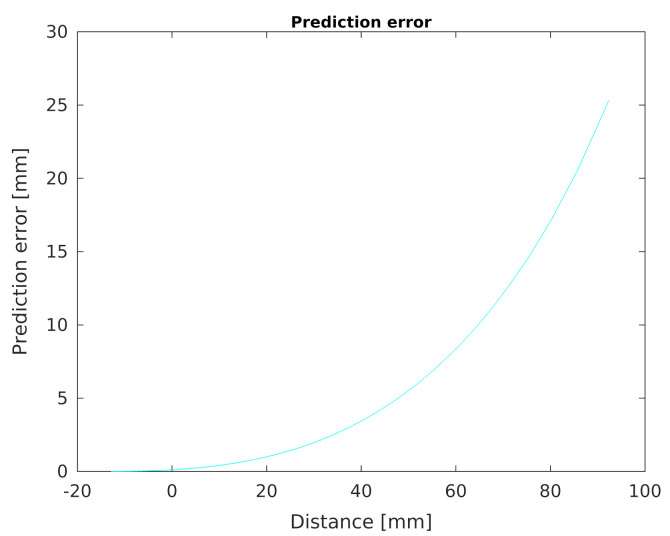
Calculated prediction error.

Finally, the prediction error chart determined by the Equation (4) as a function of the distance from the developed sensor is shown in the chart in Figure 13. It can be seen that the accuracy of determining the distance from an object changes non-linearly when this distance increases.

**Figure 14:**
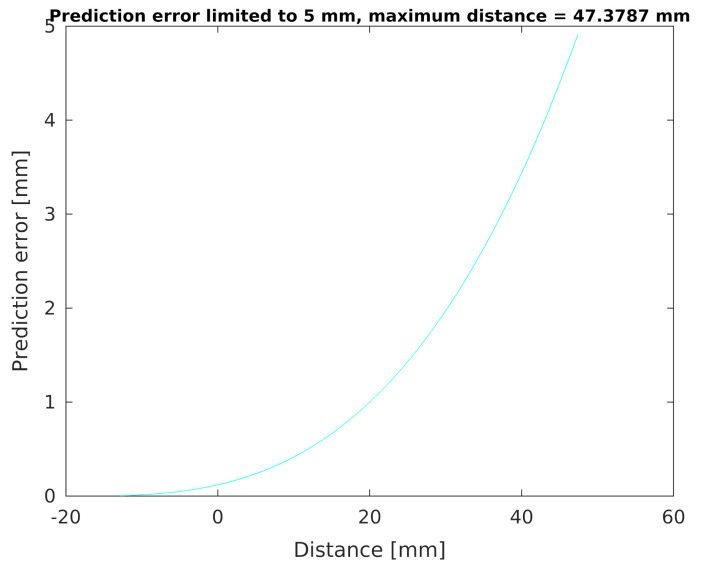
Limited prediction error.

In order to more precisely analyse the possibility of determining the distance *d* from the human hand to the developed sensor, based on the generator operating frequency $f_{g}$ measurements, an exemplary prediction error plot was also determined for values limited to the level of 5 mm (Figure 14). It can be seen that, for such limited conditions, the maximum measurement distance for the sensor is approximately 47 mm.

Regarding the second experiment, in the presented system, we set the measurement time from 2.82 to 728 μs. However, the measurement time is important when it comes to the accuracy of the results obtained. When using longer measurement times, it is possible to more accurately determine the position of the object in relation to the sensor. The use of shorter times enables the detection of the presence of an object near the sensor but does not provide precise information regarding the distance at which the object is located. For $n_{c}$ equal to 256 and the measurement acquisition time 2.82 μs, we were not even able to detect the presence of an approaching object.

## 5. Conclusions

The proposed solution enables very fast (up to 5.63 μs) detection of an object/person located near the sensor. Rapid measurements are made possible by the sensor design which reduces the overall complexity of the system. A distance measurement solution in presented system consists of only one logic gate, a resistor, and an open capacitor. The conducted tests confirmed that, for greater measurement time, the device can be used within a certain range not only to detect the proximity, but also to measure the distance to human body parts in the vicinity of the sensor. In the simplest scenario, developed device can be used for early detection of human presence in the vicinity of the machine in order to stop it. In this case, a threshold value for the signal from the distance sensor can be set. This should reduce the risk of a human-machine collision and contribute to improving the safety of human-machine cooperation. In a more complicated scenario with interactive robots (e.g., human playing Russian checkers or Chess with a robotic arm), a more advanced robot control system should be considered. Such a problem can be seen as avoiding obstacles in a dynamic environment. In this case, such an obstacle should be detected as far away from the robot as possible. The problem can be solved, for example, by re-planning the robot’s trajectory when an obstacle in the form of a human body part appears in its surroundings. We believe that, with the use of the developed device, it is possible to prepare a control system containing relatively simple rules allowing to avoid collisions with a human while attempting to perform the intended movement. This will be one of the directions of our further work. Moreover, the presented device is less susceptible to electromagnetic disturbance comparing to purely analog sensors. The capacitance of the open capacitor is directly converted into a digital signal using frequency modulation. The signal modulated in this way can be transmitted smoothly and can be easily measured with a microcontroller.

A significant advantage of the described solution is the possibility of combining the proximity sensor with a distributed touch sensor—the aforementioned robotic skin. The touch sensor electrode array may be designed to cover any shape, thereby allowing the location of a touch to be determined with high resolution. The proximity sensing described in this paper complements the touch measurements. Detecting the presence of a person/object in its vicinity does not require as high spatial resolution as measuring the location of the touch. Thus, systems with a much simpler and more reliable structure were created. Thanks to this, a greater speed of the proximity measurement system operation was achieved. It may lead to improved safety in the interaction of the robot with a human.

The described research does not exhaust the possibility of further development of this technology. The next step would be to integrate touch and proximity/distance measurement systems so that touch and proximity measurements can be measured simultaneously using the same electrode and sensor array. For this purpose, it is necessary to integrate two electronic systems, both in the digital and analog part. Another issue leading to potentially even faster proximity detection would be to design a automatic threshold detector system that indicates the presence of an object/human at a given distance, without a microcontroller—using only logical elements. Further tests of the system are also planned in order to determine the possibility of detecting objects made of various materials, as well as the influence of the changing environment on the measurement result.
